# Improving Photoelectrochemical Activity of Magnetron-Sputtered Double-Layer Tungsten Trioxide Photoanodes by Irradiation with Intense Pulsed Ion Beams

**DOI:** 10.3390/nano12152639

**Published:** 2022-07-31

**Authors:** Alshyn Abduvalov, Marat Kaikanov, Timur Sh. Atabaev, Alexander Tikhonov

**Affiliations:** 1 Physics Department, School of Sciences and Humanities, Nazarbayev University, Nur-Sultan 010000, Kazakhstan; marat.kaikanov@nu.edu.kz (M.K.); atikhonov@nu.edu.kz (A.T.); 2 Chemistry Department, School of Sciences and Humanities, Nazarbayev University, Nur-Sultan 010000, Kazakhstan; timur.atabaev@nu.edu.kz

**Keywords:** photoelectrochemical, intense pulsed ion beam, sputtering, irradiation

## Abstract

The photoelectrochemical (PEC) activity of metal oxide photoelectrodes for water-splitting applications can be boosted in several different ways. In this study, we showed that PEC activity can be significantly improved with a double-layer (crystalline-amorphous) configuration of WO_3_ thin films irradiated with intense pulsed ion beams (IPIB) of a nanosecond duration. It was found that IPIB irradiation promotes the formation of crystalline and sponge-like WO_3_ structures on the surface. Due to an increase in the active surface and light scattering in irradiated samples, photocurrent generation increased by ~80% at 1.23 reversible hydrogen electrodes (RHE).

## 1. Introduction

At the present moment, photoelectrochemical (PEC) water splitting is considered as one of the viable methods for meeting renewable energy needs. Solar-light-based hydrogen production could offer new opportunities for a variety of industries [1,2]. However, further research for efficiency improvement is needed, as it is relatively low compared with other renewable energy sources [3,4].

Tungsten trioxide WO_3_ is a metal oxide semiconductor material with a wide band gap, which can be tuned from 3.2 eV to 2.5 eV using various methods of modifications [5,6,7]. It could act as a photoanode by absorbing sunlight and generating electron–hole pairs for oxygen evolution. However, it has some drawbacks, such as photo corrosion caused by peroxo-species generation on the surface, the slow-moving kinetics of generated holes, poor charge separation [8], etc. Doping, composite material construction, heterostructured material fabrication, and surface nanostructuring may all play important roles in mitigating such disadvantages [9,10,11,12,13]. To examine the photoelectrodes associated with their morphologies, tungsten oxides in the form of nanoplates, -fibers, and -rods, and wedge- or sheet-like structures were created and tested [14,15,16,17,18]. It has also been reported that surface and morphology engineering can improve the performance of photoelectrode materials [19]. In this regard, etching and surface passivation could help to improve surface electron–hole kinetics, reduce the overpotential of thin films, and achieve an improvement in photoelectric conversion [20]. Moreover, type annealing in various atmospheres is another method that has been used to improve the photocatalytic activities of various materials because annealing has a significant impact on the structural properties of materials [21,22]. Approaches such as laser and ion irradiation may be used during the deposition or post-deposition treatments for tuning electrical, morphological, and structural characteristics [23,24,25].

In addition to the ways already stated, computational [26] and experimental strategies [27] are widely employed to increase the photoactivity of WO_3_. Recent works report on the highly enhanced photocatalytic activity of the WO_3_ layer embedded in heterostructures [28,29,30]. The proper band alignment of photoanode layers with water oxidation potentials opens a route to significantly increasing photocurrent generation because of the better charge separation in multiple-layered heterostructures. Additionally, the PEC performance of photoelectrodes can be boosted by combining 2D materials with WO_3_ [31,32], controlling the texturing of particular crystallographic facets [33,34,35], creating unassisted tandem cell structures [36], using plasmonic nanoparticles [37], and by creating oxygen vacancies [38].

In this study, we propose a new method to modify a WO_3_ photoelectrode that combines simultaneous surface nanostructuring and annealing for enhancing PEC properties. High-current-density pulsed ion beam (IPIB) irradiation promotes the formation of the sponge-like porous surface of WO_3_ with a simultaneous annealing process. Obtained samples were analyzed for morphological, structural, optical, and PEC characteristics. Hence, a novel method of surface engineering allows us to create crystalline porous sponge-like thin films with increased surface area and absorbance directly from amorphous films.

## 2. Materials and Methods

### 2.1. Sample Preparation

We prepared amorphous, crystalline, and double-layer WO_3_ samples on fluorine doped tin oxide (FTO) substrates. We washed commercial FTO (~350 nm) on glass substrates in ethanol with ultrasonic bath before deposition procedure. We used the Kurt Lesker magnetron system for the reactive DC deposition of WO_3_ using a tungsten target (W, Kurt J. Lesker, 99.95% purity, 2.00″ diameter) and holding argon/oxygen flow rates at 33/21 sccm. During the deposition, we assigned the sputtering power as 100 W, and rotated the substrate at a rate of 20 rounds per minute. We held base pressure during the deposition at 11.4 mTorr.

Initially, we deposited amorphous WO_3_ thin film samples for 45 min. It was already reported that the magnetron-sputtered WO_3_ has an amorphous nature [39]. Then, we annealed half samples at 500 °C for 20 min in air on a hot plate to obtain crystalline samples. We irradiated the prepared amorphous and crystalline samples with intense pulsed ion beam (IPIB) with ion beam current densities from 10 A/cm^2^ to 32 A/cm^2^. We performed IPIB irradiation by the INURA accelerator installed at Nazarbayev University (Nur-Sultan, Kazakhstan) [40]. INURA is a pulsed-power architecture ion accelerator with a total power consumption less than 10 kW. The peak power density of the beam at the target is up to 10 MW/cm^2^. Main parameters of the accelerator are as follows: accelerating voltage is in the range from 300 to 400 kV, total beam current is 10 kA, beam pulse length is 100 ns (FWHM). Irradiation by single pulse of 100 ns of ion beam induces superfast annealing process, with a hundred-nanosecond heating followed by a microseconds-scale cooling process. Next, we fabricated irradiated and non-irradiated double-layer WO_3_ thin films with thicknesses approx. 380 nm (top amorphous ∼120 nm, bottom crystalline ∼260 nm). Firstly, we deposited an amorphous WO_3_ layer for 30 min on two FTO substrates and annealed on a hot plate for 20 min in air to form crystalline film samples. Secondly, on the top of that crystalline film, we deposited a second layer of amorphous WO_3_ films for 15 min. Both FTO substrates were in the same conditions during the annealing and deposition processes. Then, we performed irradiation with IPIB at a current density of 6 A/cm^2^ on one of the prepared double-layer WO_3_.

We examined morphological, structural, and optical properties of irradiated samples using a Carl Zeiss Crossbeam 540 GEMINI II Scanning Electron Microscope (SEM), Rigaku Smart Lab Automated Multipurpose Grazing Incidence X-ray Diffractometer (GI-XRD) (omega–0.5), and Evolution 300 Spectrophotometer, respectively.

### 2.2. PEC Characterization

We used a three-electrode cell configuration with double-layer WO_3_ as a working electrode for the PEC testing. We measured PEC characteristics of working electrodes with Ag/AgCl (3.5 M KCl) reference electrode and Pt wire-counter electrode. We measured the photocurrent generation of samples in 0.1 M Na_2_SO_4_ (pH 7.2) electrolyte using a potentiostat CHI660E in conjunction with a PLS-EM solar simulator (100 mW/cm^2^, AM 1.5). We used the following equation to convert the measured potential to the reversible hydrogen electrode (RHE) scale:

V_RHE_ = V_Ag/AgCl_ + 0.059∗pH + 0.205 (1)

where, V_Ag/AgCl_ is the applied potential and 0.205 is the standard potential of the Ag/AgCl, KCl (3.5 M) reference electrode. We performed the electrochemical impedance spectroscopy (EIS) in a frequency range from 0.01 Hz to 10 kHz with an AC voltage amplitude of 50 mV at a DC bias of 0.68 V vs. RHE under light illumination. We used EIS Spectrum Analyzer software to obtain circuit model fitting of EIS data [41].

## 3. Results and Discussions

**Figure 1:**
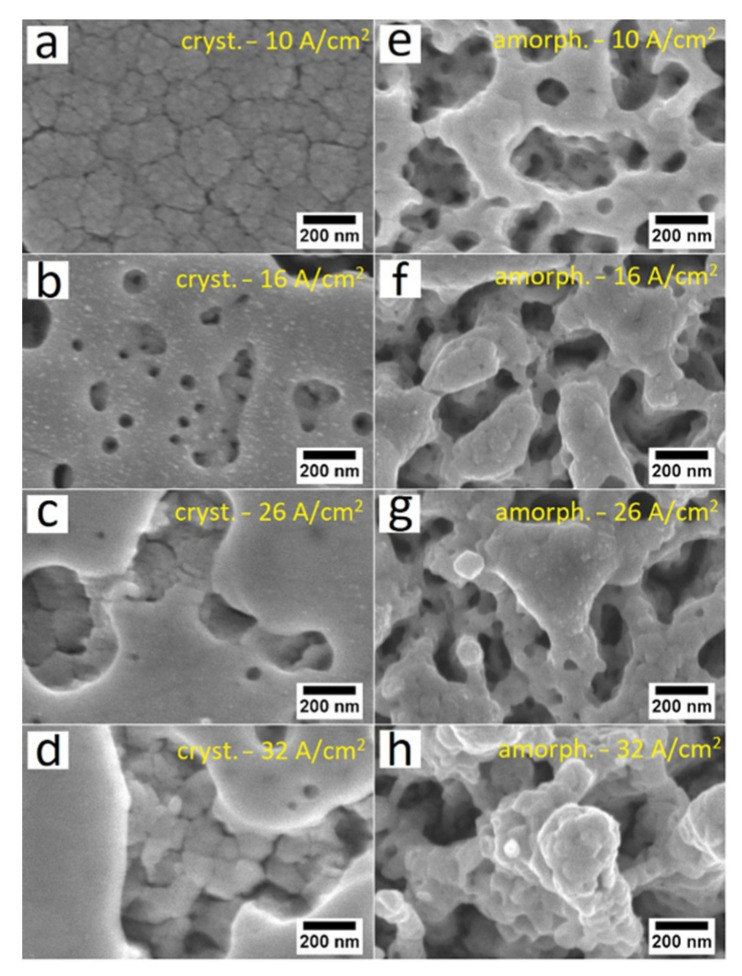
Surface SEM images of crystalline (**a**–**d**) and amorphous (**e**–**h**) WO_3_ thin films after ion beam irradiation with current densities: (**a**,**e**) 10 A/cm^2^, (**b**,**f**) 16 A/cm^2^, (**c**,**g**) 26 A/cm^2^, (**d**,**h**) 32 A/cm^2^.

Figure 1 shows surface SEM images of crystalline (a–d) and amorphous (e–h) WO_3_ thin films on an FTO/glass substrate after ion beam irradiation with current densities ranging from 10 A/cm^2^ to 32 A/cm^2^. SEM images of crystalline samples irradiated with an ion beam current density of 10 A/cm^2^ (Figure 1a) had no visible differences with SEM images of non-irradiated samples; therefore, 10 A/cm^2^ irradiation has no or very little effect on the surface morphology of crystalline WO_3_ thin film. It can be seen that irradiating with ion beam current densities of 16 A/cm^2^ and 26 A/cm^2^ (1b and 1c) drastically changed the initial thin film morphology and transformed it into a porous thin film with empty spaces opening the substrate material underneath. A high ion beam current density of 32 A/cm^2^ exposed the sample in the same way; however, the open spaces became wider. The same ion beam current densities had different effects on amorphous sample morphologies compared with crystalline samples, as seen in Figure 1e–h. After irradiation, the overall appearance of all irradiated amorphous films resembled sponge-like structures. As the ion beam current density increases from 10 to 32 A/cm^2^, the shape irregularities and sizes of the sponge-like structures become larger. It can be concluded that IPIB irradiation causes more morphological changes on amorphous surfaces than on crystalline.

**Figure 2:**
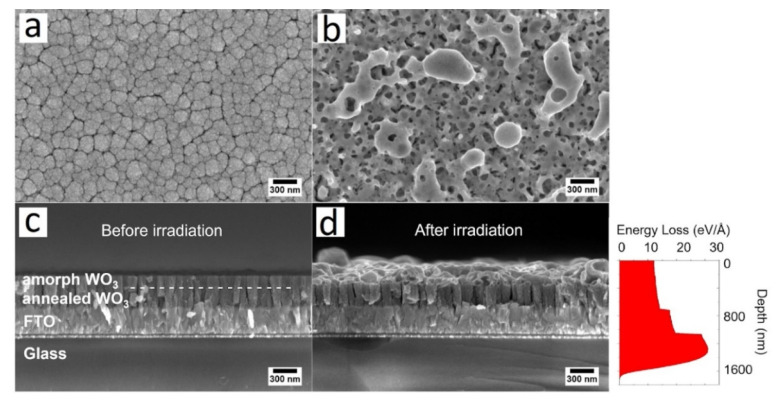
SEM images of surface of (**a**) non-irradiated and (**b**) irradiated double-layer WO_3_ at 6 A/cm^2^ and cross-sections of (**c**) non-irradiated and (**d**) irradiated double-layer WO_3_ at 6 A/cm^2^. Inset—Calculated energy loss cross-section profile of protons with initial kinetic energy of 350 keV.

We measured the PEC characteristics of amorphous and crystalline WO_3_ films irradiated with ion beams of current densities and found that increasing ion beam current density reduces the photocurrent generation of crystalline WO_3_ thin films, as shown in Figure S1. In the case of amorphous samples, photocurrent generation was not observed at any current density of irradiation. This could be attributed to an increase in various defects in films and the formation of open spaces, where FTO is accessible to electrolytes and leads to a short circuit during the photocurrent measurement. Therefore, we propose to utilize a double-layer film, an amorphous layer on top of a crystalline layer that prevents the formation of open spaces exposing the substrate. It was found that by irradiating at an ion beam current density of 6 A/cm^2^, there were no open spaces on the crystalline bottom layer, while a good sponge-like structure was observed on the top. Figure 2 shows SEM images of the surface and cross-section of double-layer WO_3_ thin films before and after irradiation with an ion beam current density of 6 A/cm^2^, i.e., confirming the nanostructuring of the top amorphous layer after irradiation.

To better understand the impact of ion beam irradiation on a WO_3_/FTO/glass target, the energy loss profile of the proton beam with an initial kinetic energy of 350 keV in the WO_3_/FTO/glass target was calculated using SRIM (the stopping and range of ions in matter) open access software (http://www.srim.org/, accessed on 25 May 2022). The results of the calculations are presented in the inset of Figure 2. Calculations revealed that a ~1600 nm ion beam range (penetration depth) in a WO_3_/FTO/glass multilayer structure, which indicates that the energy of the intense pulsed ion beam is absorbed mostly near the surface layer of the irradiating material. The absorbed energy of the intense pulsed ion beam leads to the super-fast heating of this surface layer with subsequent fast cooling, resulting in the modification of the surface morphology and crystal structure.

The surface of a non-irradiated sample has a granular microstructure; however, after ion beam irradiation, the morphology changes to a sponge-like structure with randomly distributed open spaces. Furthermore, as shown in Figure 2b, the formation of some clusters can be seen on the top layer of the irradiated sample. Figure 2c,d show a SEM cross-sectional view of the photoelectrodes before and after ion irradiation, and also show the sponge-like structure and formation of clusters. The irradiated double-layer WO_3_ thin film in Figure 2d clearly shows that the bottom crystalline layer beneath remains unchanged after the irradiation process.

**Figure 3:**
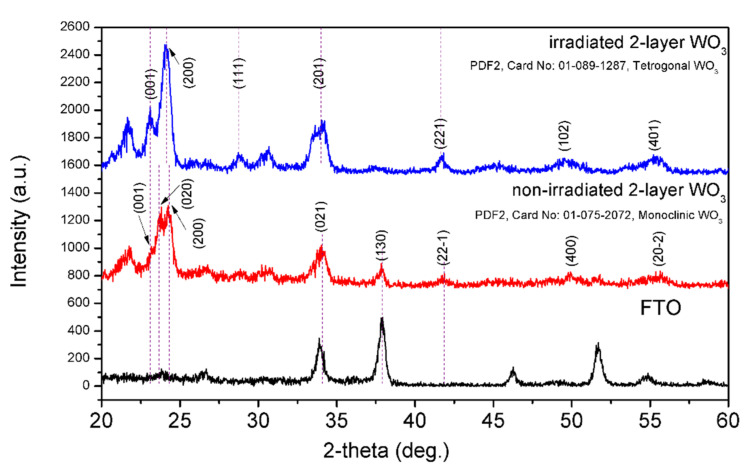
GI-XRD patterns of irradiated and non-irradiated double-layer WO_3_ thin films. Bottom plot shows GI-XRD pattern of the FTO substrate.

Grazing incidence X-ray diffraction (GI-XRD) was used to examine the structural properties of non-irradiated and irradiated double-layer WO_3_ photoelectrodes. Figure 3 shows the corresponding GI-XRD patterns. The bottom layer of the non-irradiated sample is crystalline because it was hot-plate annealed after deposition, and the top layer is amorphous. The red pattern in Figure 3 indicates the dominance of a monoclinic WO_3_ phase (PDF2, No:01-075-2072) in the bottom annealed layer. However, after irradiation, both the top amorphous layer and the bottom crystalline layer transformed into a dominating tetragonal phase (PDF2, No:01-089-1287), as shown in Figure 3 (blue pattern). The peaks of the black pattern shown on the bottom plot are from the FTO substrate and are less visible on irradiated and non-irradiated samples since the GI-XRD mode probes only a thin surface layer. Peak (020), which existed in the monoclinic phase, vanished after the ion beam irradiation. We also performed GI-XRD measurements on a one-layer irradiated amorphous sample to ensure that after a 6 A/cm^2^ ion beam irradiation, it does indeed become a predominantly crystalline tetragonal WO_3_, as shown in Figure S2. Therefore, both top and bottom layers of our double-layer sample become crystalline with a dominating tetragonal phase because of the super-fast annealing by IPIB irradiation. The GI-XRD peaks seen in Figure S2 for amorphous WO_3_ (blue pattern) are assigned to peaks from the FTO substrate. High-resolution GI-XRD patterns in Figure S4 confirm the amorphous nature of as-deposited WO_3_, and it is consistent with the literature [39].

**Figure 4:**
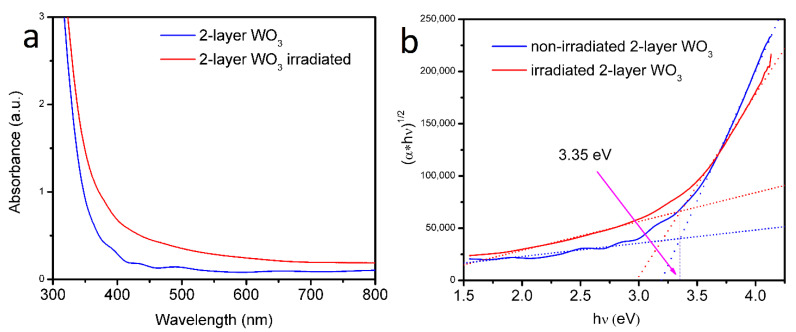
(**a**) absorption spectra and (**b**) Tauc plot of double-layer irradiated and non-irradiated WO_3_ thin films. Band gap values are indicated.

Figure 4a,b depict the absorption curves and Tauc plots of non-irradiated and irradiated double-layer WO_3_, respectively. Obviously, as depicted in Figure 4a, the irradiated double-layer sample has a higher absorbance than that of the non-irradiated sample. It can be explained by electron trapping in induced oxygen vacancies, which result after irradiation with IPIB [42]. Light scattering through the walls of sponge-like porous inclusions and light trapping on the irradiation-induced point defects of the interstitial oxygen sublattice result in increased absorption. The Tauc method was used to analyze the band gap change after the irradiation process [43]. Tauc plots for both samples in Figure 4b show the same 3.35 eV band gap before and after irradiation.

**Figure 5:**
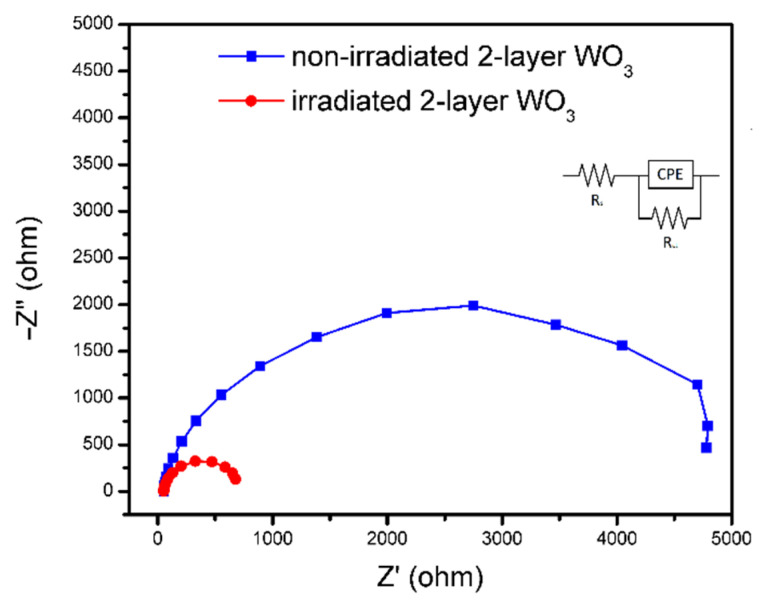
EIS Nyquist plots of non-irradiated and irradiated double-layer WO_3_.

Electrochemical impedance spectroscopy (EIS) was used to determine the charge transfer properties for irradiated and non-irradiated double-layer samples, as shown in Figure 5. It is observed that the irradiated sample shows a smaller radius of the semicircle than a non-irradiated sample, i.e., it implies that the nanostructured sample is more beneficial for the charge transfer between a photoelectrode and electrolyte. Irradiation-induced surface nanostructuring improves the charge transport properties, i.e., sponge-like structures facilitate hole migration to the WO_3_/electrolyte junction and increase the surface-active sites. Furthermore, light trapping in such a structure promotes the increased formation of electron–hole pairs through the improved photon absorption.

**Figure 6:**
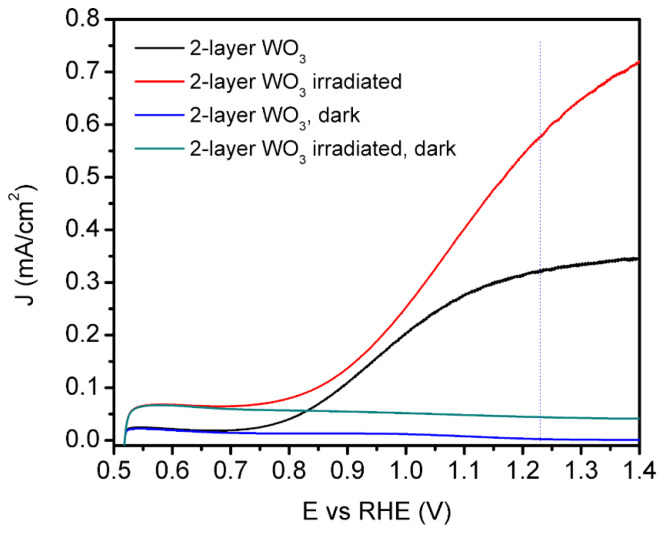
Linear sweep voltammetry curves of double-layer WO_3_ before and after the modification with IPIB of 6 A/cm^2^ ion beam current density.

The linear sweep voltammetry (LSV) curves of non-irradiated and irradiated double-layer WO_3_ films are depicted in Figure 6. Additional chopped LSV curves in Figure S3 confirm that prepared photoelectrodes show photocatalytic activity. The photocurrent of 0.32 mA/cm^2^ was measured at 1.23 RHE for bare WO_3_, and 0.58 mA/cm^2^ was measured at 1.23 RHE for irradiated WO_3_ photoelectrodes. This ~80% increase in the photocurrent density of an irradiated sample compared with a non-irradiated sample could be due to the following factors:

Firstly, an IPIB-beam-induced sponge-like structure could act as a light-trapping structure, enhancing absorption. Additionally, more absorption improvements occurred due to light capture by point defects in the oxygen sublattice [42]. Secondly, nanostructuring caused by ion bombardment may increase the active surfaces of the interface layers between the solution and photoanode. Thirdly, IPIB irradiation converts the top amorphous layer of WO_3_ into a crystalline phase. It was already proved that crystalline WO_3_ has an advantage over amorphous WO_3_ in PEC performance [44]. Accordingly, IPIB irradiation could be considered as one of the methods for creating porous and crystalline materials with improved PEC activity.

In general, irradiation with ion beams is widely used for material modifications [45]. As a result of ion beam impact, a series of effects, such as temperature increases, ion doping, and atomic displacement, cause changes in the target material. Continuous ion beams with low current densities are widely used for tailoring the electronic, morphological, and structural properties of target materials. For example, in Ref. [24], WO_3_ films were irradiated with 190 keV Ar+ ion beams with current densities ranging from 1 to 2 μA cm^−2^. It was shown that morphological changes (formation of pores) appear when the fluence of the Ar+ ion beam reaches 10^17^ ions·cm^−2^. For the IPIB, the irradiation of materials with very short and powerful pulses causes a very fast and intense energy deposition onto the material’s surface. For instance, we use 100 ns-duration IPIB with a typical fluence of ions in a single pulse of about 10^12^ protons·cm^−2^, which provides a rate of ions to the target up to 10^19^ protons cm^−2^ s^−1^ and corresponds to the power density of 1 MW/cm^2^. This high-power density is deposited into the thin surface layer, leading to a fast temperature increase. It can be used with a higher beam intensity, even in high-energy-density physics, to ignite warm-dense-matter states [46]. In practical applications, the fast-heating annealing effect can be used for the modification of the near-surface layer of bulk targets [47]. The main result in our paper is the demonstration of the applicability of IPIB with appropriate current densities for the useful structural modification of photoactive thin films based on WO_3_. Due to the fast injection of ions, the required fluence of IPIB (10^12^ ions·cm^−2^) is significantly lower when compared with a continuous ion beam (higher than 10^16^ ions·cm^−2^).

## 4. Conclusions

In summary, we introduced a method for creating porous double-layer WO_3_ films with improved PEC activity. Irradiation of double-layer WO_3_ films with a single pulse of IPIB leads to a working electrode with a sponge-like crystalline surface layer with improved light absorption and charge transport properties. We revealed that the photocurrent was increased to ~80% because of the increase in the active surface and enhancement of charge transport characteristics. Future research can address the stability issues of irradiated WO_3_ films. We believe that our method can be utilized and further studied to boost photoactive materials by IPIB irradiation.

## Data Availability

Not applicable.

## Supplementary Materials

The following supporting information can be downloaded at: https://www.mdpi.com/article/10.3390/nano12152639/s1. Figure S1: LSV curves of irradiated crystalline WO_3_ thin films with increasing ion beam current density. Black arrows indicate that photocurrent generation reduces with increasing ion beam current densities; Figure S2: GI-XRD patterns of initially amorphous WO_3_ thin film before and after irradiation with 6 A/cm^2^ ion beam current density; Figure S3: chopped linear sweep voltammetry curves of double-layer WO_3_ before and after the modification with IPIB of 6 A/cm^2^ ion beam current density; Figure S4: high-resolution GI-XRD patterns of amorphous WO_3_ and FTO substrate.
