# Supplementary material for: Improving Photoelectrochemical Activity of Magnetron-Sputtered Double-Layer Tungsten Trioxide Photoanodes by Irradiation with Intense Pulsed Ion Beams
Source: Nanomaterials (Basel). 2022 Jul 31;12(15):2639. doi: 10.3390/nano12152639 (PMC9370333; doi:10.3390/nano12152639)
Supplement: Supplementary file 1 [file nanomaterials-12-02639-s001.zip › nanomaterials-1812751-supplementary.pdf]

nanomaterials

Supplementary materials

Improving Photoelectrochemical Activity of Magnetron-Sputtered Double-Layer Tungsten Trioxide Photoanodes by Irradiation with Intense Pulsed Ion Beams

Alshyn Abduvalov [1,*], Marat Kaikanov [1], Timur Sh. Atabaev [2] and Alexander Tikhonov [1]

[1] Physics Department, School of Sciences and Humanities, Nazarbayev University, Nur-Sultan 010000, Kazakhstan; marat.kaikanov@nu.edu.kz (M.K.); atikhonov@nu.edu.kz (A.T.)
[2] Chemistry Department, School of Sciences and Humanities, Nazarbayev University, Nur-Sultan 010000, Kazakhstan; timur.atabaev@nu.edu.kz
* Correspondence: alshyn.abduvalov@nu.edu.kz; Tel.: +7-747-583-11-92

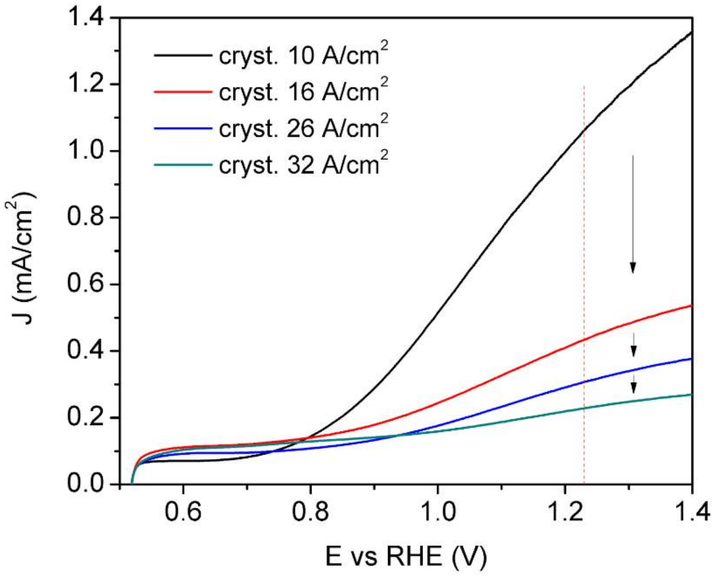

Figure S1. LSV curves of irradiated crystalline WO_3 thin films with increasing ion beam current density. Black arrows indicate that photocurrent generation reduces with increasing ion beam current densities.

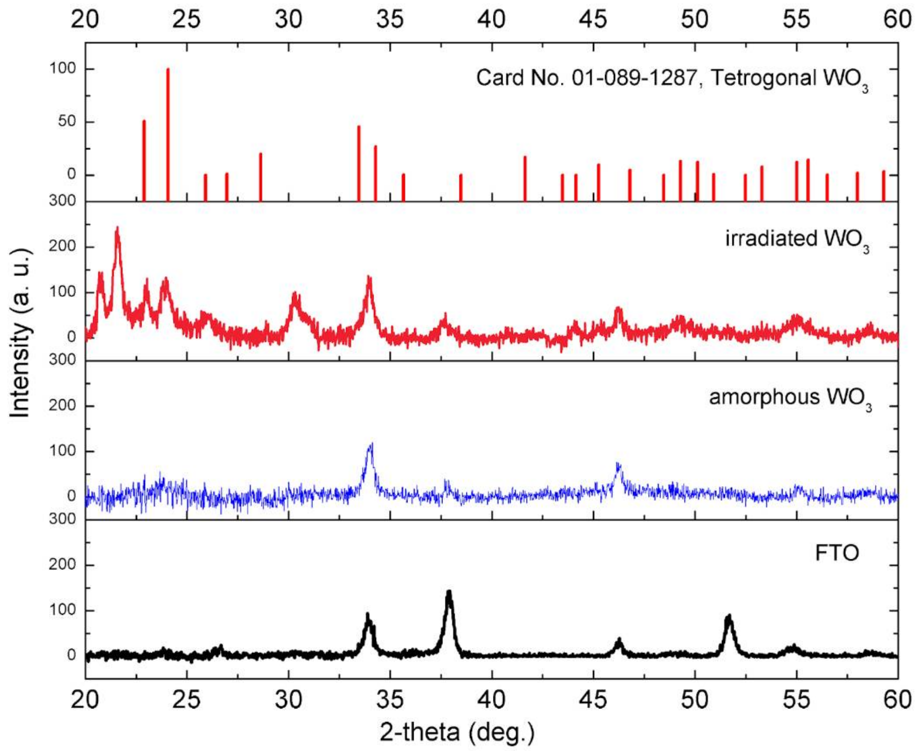

Figure S2. GI-XRD patterns of initially amorphous WO_3 thin film before and after irradiation with 6 A/cm^2 ion beam current density.

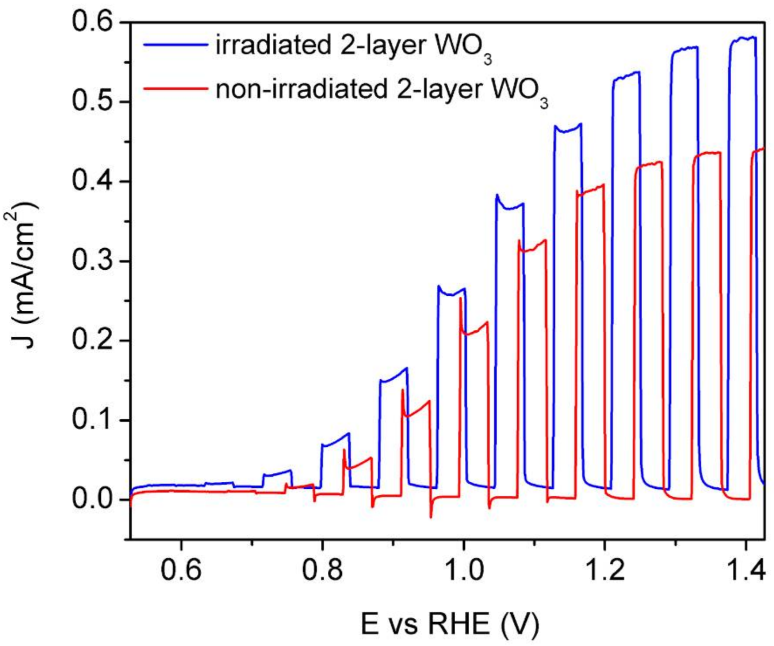

Figure S3. Chopped linear sweep voltammetry curves of double-layer WO_3 before and after modification with IPIB of 6 A/cm^2 ion beam current density.

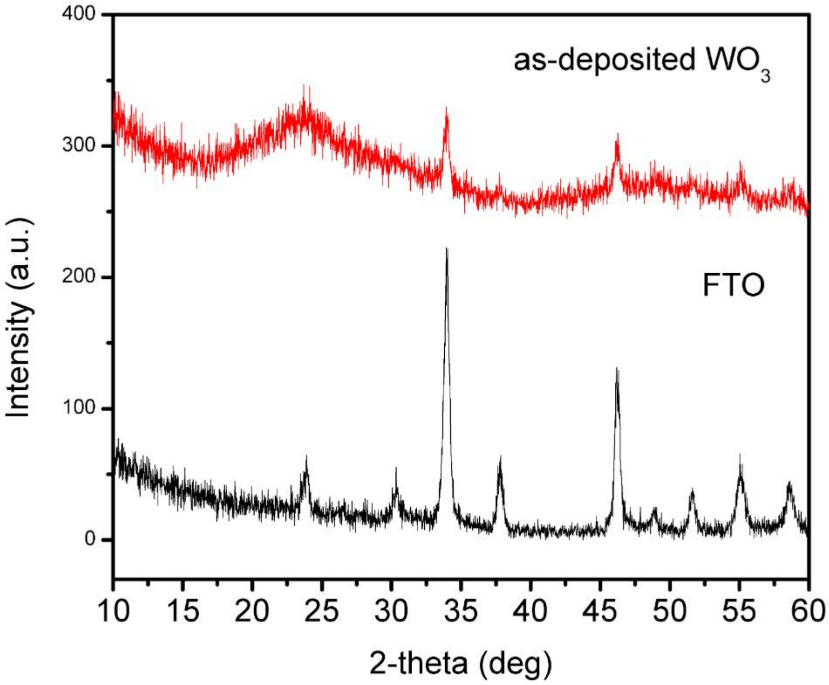

Figure S4. High resolution GI-XRD patterns of amorphous WO_3 and FTO substrate
